# Cardiac ischemia and reperfusion in mice: a comprehensive hemodynamic, electrocardiographic and electrophysiological characterization

**DOI:** 10.1038/s41598-023-32346-5

**Published:** 2023-04-07

**Authors:** Lukas Clasen, Stephan Angendohr, Stefanie Becher, Benedikt Bartsch, Stephan Enkel, Christian Meyer, Malte Kelm, Hisaki Makimoto, Nikolaj Klöcker

**Affiliations:** 1 Department of Cardiology, Pulmonary and Vascular Diseases, Medical Faculty and University Hospital Düsseldorf, Heinrich-Heine-University, Düsseldorf, Germany; 2 Division of Cardiology, Angiology, Intensive Care Medicine, EVK Düsseldorf, cNEP, Cardiac Neuro- and Electrophysiology Research Consortium, Düsseldorf, Germany; 3 Institute of Neural and Sensory Physiology, Medical Faculty and University Hospital Düsseldorf, Heinrich-Heine-University, Düsseldorf, Germany; 4 Cardiovascular Research Institute Düsseldorf (CARID), Medical Faculty and University Hospital Düsseldorf, Heinrich-Heine-University, Düsseldorf, Germany; 5 Present Address: Department of Cardiology, Rhythmology and Angiology, Josephs-Hospital Warendorf, Academic Teaching Hospital, University of Münster, Warendorf, Germany; 6 Present Address: Department of Internal Medicine II, Heart Center Bonn, University Hospital Bonn, Bonn, Germany

**Keywords:** Cardiovascular biology, Cardiovascular diseases, Arrhythmias

## Abstract

Malignant ventricular arrhythmias (VA) after acute myocardial infarction remain a major threat. Aim of this study was to characterize the electrophysiological and autonomic sequelae of cardiac ischemia and reperfusion (I/R) in mice during the first week post incident. Left ventricular function was serially assessed using transthoracic echocardiography. VA were quantified by telemetric electrocardiogram (ECG) recordings and electrophysiological studies on the 2nd and 7th day after I/R. Cardiac autonomic function was evaluated by heart rate variability (HRV) and heart rate turbulence (HRT). Infarct size was quantified by planimetric measures. I/R caused significant myocardial scarring and diminished left ventricular ejection fraction. The ECG intervals QRS, QT, QT_c_, and JT_c_ were prolonged in I/R mice. Both spontaneous VA scored higher and the inducibility of VA was raised in I/R mice. An analysis of HRV and HRT indicated a relative reduction in parasympathetic activity and disturbed baroreflex sensitivity up to 7 days after I/R. In summary, during the first week after I/R, the murine heart reflects essential features of the human heart after myocardial infarction, including a greater vulnerability for VA and a decreased parasympathetic tone accompanied by decelerated depolarization and repolarization parameters.

## Introduction

Sudden cardiac death (SCD) due to ventricular arrhythmias (VA) accounts for about half of the mortality of ischemic heart disease, which remains the predominant single cause of death in the Western world^1^. With the development of early reperfusion therapy including thrombolysis and primary percutaneous coronary interventions (pPCI) during last decades, the acute mortality of myocardial infarction (MI) has declined to well below 5%^2^. However, survivors of acute MI remain at high risk of premature death due to lethal arrhythmias^3^. The management of VA after MI still poses a major challenge as both risk assessment and pharmacological treatment options are largely limited.

Myocardial damage after ischemia and reperfusion (I/R) differs from that after no-reflow ischemia owing, among others, to a sterile injury and inflammatory response that adds to the primary tissue loss^4^. While the duration of ischemia is an obvious determinant of infarct size (IS), the latter may further be modulated by early cardioprotective interventions. The size of infarction may well set the clinical outcome as it eventually relates to both ventricular dysfunction and the occurrence of life-threatening VA^5^. I/R injures myocardial autonomic neurons beyond the necrotic infarct zone within the area at risk^6^. It has also been reported that cardiac autonomic control as assessed by heart rate variability (HRV) may modulate the incidence of VA after MI^7^. In the infarcted heart, sympathetic stimuli increase the dispersion of repolarization compromising the electrical stability of the ventricles^8^. Moreover, a disturbed baroreflex as appreciated by disordered heart rate turbulence (HRT) was independently associated with death after MI^9^.

Mouse models have proven invaluable for gaining insight into the contribution of specific genes and signal transduction pathways to cardiovascular pathophysiologies^10^. Two in vivo models of only chronic cardiac ischemia by permanent ligation of the left anterior descending (LAD) coronary artery have been characterized in mice. Gehrmann et al. described an ECG pattern in wildtype mice characteristic for myocardial ischemia within 14 days, exhibiting a diminished R wave amplitude, early ST segment elevation, and later development of an inverted T wave and a significant Q wave^11^. Korte et al. reported QRS broadening and prolonged repolarization in sedated animals after 11 weeks of chronic cardiac ischemia^12^. Contradictory to the observation in humans, however, both models of chronic ischemia did not exhibit any cardiac autonomic dysfunction. The authors explained this by a higher basal sympathetic tone in mice compared to humans. Very recently, sympatho-excitation and parasympathetic withdrawal were demonstrated in transgenic mice after permanent LAD occlusion^7^.

Here, we sought to establish a serial electrophysiological in vivo characterization of arrhythmia in reperfused MI in mice, which much more closely resembles today’s clinical situation after catheter intervention in acute MI. By combining telemetry, electrophysiological stimulation, and echocardiography, we thoroughly characterized the functional properties of the murine heart during the first week after I/R. The presented mouse model will provide a starting point for investigating the mechanisms underlying ventricular arrhythmogenesis in ischemic heart disease eventually contributing to improved risk assessment and prevention of SCD after MI also in humans.

## Materials and methods

### Ethical statement

All experiments were in accordance with the established guidelines ARRIVE 2.0 (Du Sert 2020) and had been approved by the Animal Ethics Committee of the North Rhine-Westphalian State Agency for Nature, Environment and Consumer Protection, Germany (project numbers: 84-02.04.2013.A460, 81-02.04.2019.A204). All the methods were performed in accordance with relevant guidelines and regulation.

### Animals and study protocol

Male C57BL/6J wild type mice (14–16 weeks of age, Janvier, Le Genest Saint Ilsle, France) were subjected to I/R (n = 20) and sham operation (n = 16) as described below. Continuous electrocardiogram (ECG) monitoring was conducted by subcutaneously implanted telemetric devices one week before baseline recording in I/R (n = 10) and sham mice (n = 6). By serial transthoracic echocardiography, left ventricular (LV) function was assessed in all animals. Finally, an invasive electrophysiological study (EPS, details below) was performed 7 days after the index procedure, with a subset of n = 6 animals of each group receiving EPS already after 2 days. Hearts were excised for histologically determining the size of ischemic injury after EPS (for details see below). For peri- and postoperative analgesia mice received buprenorphine s.c. (0.1 mg/kg bodyweight). After final in vivo electrophysiological study, mice were exsanguinated under deep sedation with ketamine (100 mg/kg bodyweight) and xylazine (10 mg/kg bodyweight).

### Myocardial ischemia–reperfusion (I/R)

Analgosedated mice (isoflurane 2 Vol.%, buprenorphine 0.1 mg/kg bodyweight s.c.) were intubated and ventilated under pressure control (MiniVent Type 845, Hugo Sachs Elektronik Harvard Apparatus GmbH). Body core temperature was continuously controlled at 37 °C with a rectal probe. Peri-operatively, surface electrogram was recorded. Under sterile conditions, the heart was cautiously exposed under retraction of the third and fourth rib. The pericardium was dissected. Occlusion of the left anterior descending coronary artery (LAD) was performed for 45 min followed by continuous reperfusion as described before^13^. Successful myocardial ischemia was documented by visual blanching of the apex and ECG ST segment elevation. In sham operations, the procedure was the same albeit coronary occlusion. Subsequently, the chest was closed. During post-operative recovery, the mice received analgesia with buprenorphine (0.1 mg/kg bodyweight, s.c.).

### Determining factors of I/R injury reproducibility

The procedure requires a well-trained experimenter with a treasure trove of experience of > 50 surgeries in advance. Strict control of the body core temperature of the animal at 37.0–37.5 °C is prerequisite for consistent myocardial I/R injury. Lower body core temperatures will reduce infarct size (IS). Therefore, the surface of the pre-heated operating table should be protected from convective loss of heat by air sealing, e.g. covering the setup with an inverted cage. Additionally, infrared lamp illumination may help to retain a stable body core temperature of the animal. Ventilation settings should be standardized, e.g. 140 breaths/min, 250 µl tidal volume and 10–12 mbar pressure, mixture of oxygen at 0.1 l/min and room air at 0.2 l/min. Volatile anesthesia should be titrated at 2 Vol.% before induction of ischemia and maintained throughout the experiment. LAD course and branching is visualized underneath the tissue surface by a binocular microscope a few mm beneath the left atrial appendage. The transient occlusion of the proximal LAD is performed by ligation, using a 7-0 prolene suture that is secured with a polyethylene tube of 3 mm under a defined tractive force putting a free-hanging weight of 1 g on each suture end. The quality of I/R injury may be validated by determining the area at risk (AAR) and the infarct area.

### Telemetric electrocardiogram (ECG) recordings

Mice were anesthetized by intraperitoneal injection of ketamine (45 mg/kg bodyweight) and xylazine (10 mg/kg bodyweight). Telemetric ECG devices (ETA-F10, weight 1.6 g; Data Sciences International, MC s’Hertogenbosch) were implanted in the abdominal cavity. Leads were placed subcutaneously at the right and left chest in position Einthoven I/II. Postoperatively, wound healing was allowed for at least 10 days.

A 24 h ECG was recorded at a sampling rate of 1 kHz for baseline and the days 0, 1, 3, and 7 after I/R detecting spontaneous VA in unrestrained and freely moving mice (LabChart V8.0, ADInstruments). ECGs were analyzed manually by two trained examiners under supervision of a clinical electrophysiologist. ECG intervals were measured from a signal average of 250 beats in a period of 3 min at 5–6 p.m. and analyzed. Parameters included SCL (ms), HR (bpm), P duration (ms), PR (ms), QRS (ms), QT (ms), QTc (ms), JT (ms), JTc (ms), Tpeak-Tend (ms) and R Amplitude (mV). Rate correction for QT and JT was performed as described for mice QTc = QT/(RR × 100)^1/2^. The ventricular depolarization interval is represented by the QRS complex and ventricular repolarization is read out by QT, JT, QT_c_, and JT_c_, respectively. To quantify spontaneous VA during telemetric ECG monitoring, we utilized a recently reported score weighting VA by quantity and duration^14^. VA score was given as mean per hour or minute within the time period, as indicated.

Heart rate variability (HRV) was analyzed from telemetric ECG monitoring (HRV 2.0 module, LabChart V8.0, ADInstruments). A rather noise-free interval of 3 min at 5–6 p.m. was selected. Heartbeats were verified in the beat classifier view. Parameters for time domain analysis were mean normal-to-normal (NN, ms), standard deviation of all NN intervals (SDNN, ms), square root of the mean square of successive differences of adjacent NN intervals (RMSSD, ms) and the percentage of normal consecutive NN intervals differing > 6 ms (pNN6, %)^7^. For frequency domain parameters, 1024 data points were calculated with the Lomb-Scargle periodogram. Ectopics were excluded. Frequency bands comprised high frequency (HF 1.5–5 Hz), low frequency (LF 0.15–1.5 Hz), and very low frequency (VLF 0–0.15 Hz) as defined for mice. The power (ms^2^) in these bands was calculated, and LF and HF were expressed as normalized units (nu). Finally, the LF/HF ratio was determined.

### Echocardiography

Transthoracic Doppler high-resolution echocardiography (Vevo 2100, Visual Sonics) with a MS-400 transducer (18–32 MHz) was performed in mice sedated by isoflurane^13^. LV volume, ejection fraction (EF, %), fractional shortening (FS, %), stroke volume (SV, µl) and cardiac output (CO, ml/min) were determined by tracing end-diastolic and end-systolic areas in longitudinal axis. Left ventricular wall thickness was measured in M-mode at the mid-ventricular level.

### Electrophysiological study (EPS)

EPS was performed at 2 days and 7 days after I/R and sham operation, respectively. Mice were sedated by isoflurane inhalation (1.6 Vol.%, induction period 2.5 Vol.%) and were placed in a supine position on a temperature-controlled operating table to maintain the body core temperature at 37 °C. A six limb lead surface ECG was obtained with prick electrodes (Octal BioAmp ML138, ADInstruments) connected to a PowerLab 8/30 (ADInstruments). In lead I, every 50 beats of a period of 1 min were averaged and analyzed manually as described before^14^. Measurements comprised HR (bpm), CL (ms), P-wave duration (ms), PR interval (ms), QRS interval (ms) and rate corrected QT interval (ms). QRS fragmentation (fQRS) is characterized by an additional R wave or notching of the S wave, or > 1 additional R waves in 2 leads (Fig. S2).

Endocardial EPS was conducted using a 2.0 F (0.67 mm) octapolar electrophysiology catheter (0.5 mm electrode spacing; CIBer Mouse, NuMed) as described earlier^14^. The catheter was positioned into the right ventricle via the right jugular vein under ECG monitoring. Ventricular stimulation was achieved with an external stimulator (STG 4002, Multi Channel Systems MCS GmbH, Reutlingen Germany) by rectangular impulses of 1 ms delivered at twice the pacing threshold voltage (MC Stimulus II, Multi Channel Systems MCS GmbH, Reutlingen Germany). Data was analyzed with LabChart 8.0 software suite (ADInstruments). Single premature ventricular complexes (PVC) were elicited by QRS sensed pacing at 50%, 60%, and 70% of SCL, 10 beats each, in order to study HRT. TO was calculated using the following formula TO = (RR1 + RR2) − (RR-2 + RR-1)/(RR-2 + RR-1) * 100 [%]. TS is defined as the maximum positive regression slope assessed over any five consecutive sinus rhythm RR intervals within the first 15 RR intervals after PVC. Physiologically, the initial acceleration of the sinus rate after the PVC is reflected by a negative TO, whereas deceleration of the sinus rate is reflected by a positive TS^9^. Ventricular refractory period (VRP) was obtained by S1S2 stimulation with trains of 8 S1-stimuli (CL 100 ms, 90 ms, 80 ms) and decremental coupling of S2 intervals (initial coupling interval 60 ms, 2 ms decrement). VRP is an endocardial measure of local ventricular electrical conduction. Inducibility of VA was tested using different programmed electrical stimulation (PES) protocols: (1) conventional: two (S2S3) and three (S2–S4) extrastimuli (initial coupling interval 60 ms, 2 ms decrement) after a train of eight S1-stimuli (CL 100 ms) and a consecutive stimulus 20 ms above the refractory period. (2) miniburst (MB): three (S4) to ten (S11) extrastimuli (initial coupling interval 60 ms, 2 ms decrement) after a train of twenty S1-stimuli (CL 100 ms). (3) burst: S1S1 at 50 ms, 40 ms, 30 ms, 20 ms and 10 ms over a period of 1 s. The order of the induction protocols was alternated between experiments, e.g. animal 1: 1–2–3, animal 2: 2–1–3, etc. After isoproterenol injection (1 µg/g i.p.), the alternating induction protocols were repeated with direct progression to S11-stimulation during the MB protocol skipping S4–S10 to avoid a prolonged study. To quantify VA induced by EPS, we utilized a recently reported score, weighting arrhythmias by quantity and duration: single PVC (1 point), couplets (3 points), triplets (4 points) and VT (≥ 4 ventricular complexes = 5 points, ≧ 1 s = 6 points)^14^.

### Assessment of infarct size

Histological processing documented the dynamic genesis of cardiomyocyte injury and resulting scar formation. The necrotic core of the initial ischemic lesion is surrounded by a bordering area at risk (AAR) caused by reperfusion injury encompassing different phases of sterile inflammation, which fail to rescue all injured myocardium over time. Therefore, the area of initial I/R injury and the resulting infarcted myocardium were determined at 2 days and 7 days after I/R, respectively.

After 2 days, the heart was excised, the LAD was re-occluded in the same location, and 1% Evans Blue dye was injected into the aortic root to delineate the infarcted area (INF) and AAR from the myocardium not-at-risk. The heart was then cut into 1 mm slices. Viable and necrotic sections of the AAR were identified by incubating the heart in 1% 2,3,5-triphenyltetrazolium chloride (TTC) at 37 °C for 5 min. The areas of INF (white), AAR (red), and non-ischemic LV (blue) were assessed by means of computer-assisted planimetry (Diskus software; Hilgers, Königswinter, Germany). The observer was blinded for sample identity^13^. The infarcted area at 2 days is given as AAR/LV and INF/AAR.

After 7 days, the excised hearts were fixed in 4% formaldehyde, dehydrated in an ascending alcohol series, cleared with Roticlear® (Carl Roth GmbH+Co. KG), and transferred into heated paraffine (Sakura Tissue-Tek VIP E300 Tissue Processor, Staufen Germany). Embedding was performed using a modular tissue embedding center (Leica EG1150 H, Leica Biosystems, Wetzlar Germany). Cross sections of the heart were obtained at 5 µm thickness (10 per mouse ventricle, 250 µm intervals) and stained with Gomori’s one-step trichrome staining (GTS). Scar size was assessed by computer-assisted planimetry (Diskus software; Hilgers, Königswinter, Germany) and expressed in percentage of LV volume.

### Statistical analysis

Normally distributed data are presented as mean ± standard deviation. The Student’s *t*-test, one-way or two-way analysis of variance (ANOVA) were performed when appropriate to test for statistical significance. For non-normally distributed data, the Wilcoxon test or Kruskal–Wallis test were applied. The number of asterisks indicate different levels of statistical significance with *p < 0.05; **p < 0.01; and ***p < 0.001). All analyses were performed using GraphPad Prism software v6.01 (GraphPad Software, San Diego, CA).

## Results

### Procedures and mortality

All sham-operated mice and 18 out of 20 I/R mice survived throughout the study. Hence, the overall survival rate was 94%. Of the two mice dead, one died during coronary artery ligation with a documented progressive atrioventricular block, the other one died during recovery from anesthesia without further ECG monitoring. The mean age at the time of surgery was 15.5 ± 0.5 weeks and 15.3 ± 0.8 weeks for I/R and sham mice, respectively. The mean body weight at the time of EPS was 27 ± 2 g for I/R mice and 28 ± 2 g sham mice.

### I/R impairs left ventricular function

**Figure 1:**
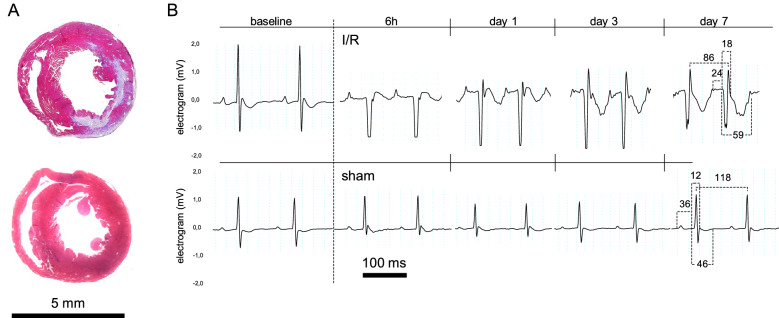
Alterations in surface electrogram after I/R in mice. (**A**) Gomoris trichrome staining of transverse sections at the mid-cavity level of the left ventricle from a mouse 7 days after I/R yielding an infarction size of 33% (upper panel) and a sham control mouse (lower panel). (**B**) Representative telemetric electrogram recordings from a mouse after I/R (upper panel) and after sham operation. Duration of indicated intervals is given in ms. Note a marked shortening of the RR interval and prolongation of the QRS and QT interval 7 days after I/R. QRS morphology often presented with negatively deflected fragmentation after I/R.

In order to validate the mouse I/R model, TTC staining was used to quantify the area-at-risk (AAR) and the infarcted area (INF) at 2 days post I/R, whereas GTS determined fibrotic infarct size (IS) at 7 days post I/R, respectively (Fig. 1). 45 min I/R caused significant myocardial scar lesions. AAR given as a fraction of left ventricular area (AAR/LV) at 2 days after I/R was 48.2 ± 7.0%. INF given as a fraction of AAR (INF/AAR) at 2 days after I/R was 57.1 ± 5.8%. IS given as a fraction of left ventricle area (IS/LV) at 7d after I/R was 35.9 ± 6.1%.

The echocardiographic evaluation demonstrated that the cardiac output (CO), stroke volume (SV), fractional shortening (FS) and ejection fraction (EF) were significantly reduced after I/R as compared to sham (Table 1). The endsystolic and enddiastolic volume were enlarged after I/R. Moreover, serial assessment demonstrated a progression in the impairment of LV hemodynamics during the first week with an apparent loss of cardiac contractility being insufficiently compensated by the intermittent increase in HR. Thus, CO declined from − 28% at 2 days to − 36% at 7 days after I/R as compared to baseline. The LVEF was impaired throughout the whole observation period after I/R contrasting sham mice (BL: 54.7 ± 3.5% vs 54.7 ± 2.8%, n.s.; 2 days: 31.4 ± 2.5% vs 52.4 ± 2.4%, *p* < 0.001; 7 days: 30.6 ± 2.0% vs 56.9 ± 3.5%, *p* < 0.001). Finally, I/R mice showed increased chamber diameters as compared to sham-operated mice (e.g. at 7 days, enddiastolic volumes: 75.6 ± 3.0 µl vs 67.8 ± 5.9 µl, *p* = 0.008; endsystolic volumes: 52.5 ± 2.6 µl vs 29.3 ± 4.1 µl, *p* < 0.001).

**Table 1:** Systolic left ventricular function is impaired during the first week after I/R.

Echocardiographic parameters	Sham baseline	I/R baseline	Sham 2 days	I/R 2 days	Sham 7 days	I/R 7 days	*p*
	n = 12	n = 16	n = 6	n = 6	n = 6	n = 10	I/R vs. sham
Heart rate (bpm)	476 ± 36	480 ± 47	536 ± 33	522 ± 45	466 ± 43	492 ± 51	0.830/0.544/0.215
Cardiac output (ml/min)	17.6 ± 1.6	17.7 ± 2.1	19.2 ± 2.2	12.7 ± 1.9***	17.9 ± 2.1	11.4 ± 1.4***	0.916/< 0.001/< 0.001
Stroke volume (µl)	37.0 ± 2.0	36.8 ± 2.5	35.7 ± 2.7	24.3 ± 2.2***	38.5 ± 3.3	22.9 ± 1.5***	0.902/< 0.001/< 0.001
Fractional shortening (%)	13.5 ± 1.7	13.3 ± 1.1	12.8 ± 1.4	7.1 ± 0.7***	13.1 ± 2.2	7.7 ± 0.7***	0.724/< 0.001/< 0.001
Ejection fraction (%)	54.7 ± 2.8	54.7 ± 3.5	52.4 ± 2.4	31.4 ± 2.5***	56.9 ± 3.5	30.6 ± 2.0***	0.987/< 0.001/< 0.001
Endsystolic volume (µl)	30.7 ± 3.8	30.6 ± 3.9	32.4 ± 1.9	52.9 ± 2.8***	29.3 ± 4.1	52.5 ± 2.6***	0.949/< 0.001/< 0.001
Enddiastolic volume (µl)	67.7 ± 5.0	67.5 ± 4.8	68.1 ± 3.1	77.2 ± 3.1***	67.8 ± 5.9	75.6 ± 3.0**	0.913/< 0.001/0.008

Data are given as mean ± SD. Statistical significance of differences between I/R and sham control was assessed by unpaired *t*-test analysis (two-tailed comparison, **p < 0.01; ***p < 0.001).

### I/R associates with delayed ventricular signal formation and increased arrhythmia vulnerability

A total of 16 mice were implanted a telemetric ECG device with subsequent successful ECG recordings over the whole study period. 30 out of 32 mice successfully underwent an EPS procedure. Right ventricular catheterization via jugular venous access failed in two I/R mice at 7 days, most likely due to postoperative thoracic adhesions. Complete 6-lead ECG data sets were obtained from 22 mice (63%).

#### Prolonged ventricular depolarization and repolarization after I/R

After I/R, characteristic changes of the electrogram were observed (Fig. 1 and Fig. S2). ECG analysis from ambulatory telemetry recordings demonstrated an increase in HR until 1 days after I/R and then persisting higher HR as compared to baseline until 7 days after I/R (*p* = 0.005). Also, prolonged cardiac de- and repolarization were observed in I/R mice compared to sham-operated mice during the first week after I/R (Fig. 1). Ventricular depolarization assessed by QRS width was delayed by 33% at 7 days after I/R compared to baseline. Likewise, QRS was broadened compared to sham-operated mice (15.8 ± 1.9 ms vs 12.1 ± 1.1 ms, *p* < 0.001) (Fig. 1, Table 2). In addition, repolarization was disturbed after I/R compared to sham-operated mice reflected by QT, rate-corrected QT, and rate-corrected JT intervals (7 days after I/R: QT: 65.5 ± 6.1 ms vs 56.5 ± 4.3 ms, *p* < 0.001; QT_c_: 64.1 ± 4.3 ms vs 51.1 ± 4.6 ms, *p* < 0.001; JT_c_: 48.7 ± 3.6 ms vs 40.4 ± 4.5 ms, *p* < 0.001) (Table 2). However, T_peak_–T_end_ was not prolonged in I/R mice, just as was the case for atrial or atrioventricular conduction (Table 2). VRP correlated with rate-corrected QT and rate-corrected JT intervals (linear regression analysis of VRP on on QT_c_: y = 0.4430*x + 48.57, r^2^ = 0.6579, *p* = 0.015; of VRP on JT_c_: y = 0.3969*x + 35.17, r^2^ = 0.7105, *p* = 0.009). VRP, QRS, and JT_c_ did, however, not correlate with increasing IS (linear regression of VRP on IS, y = 0.3639*x + 20.93, r^2^ = 0.1520, *p* = 0.265; linear regression of QRS on IS, y = − 0.007433*x + 15.81, r^2^ = 0.0006309, *p* = 0.952; regression line of JTc on IS, y = 0.1868*x + 41.89, r^2^ = 0.1711, *p* = 0.308).

**Table 2:** Heart rate increases with prolonged ventricular depolarization and repolarization after I/R.

Electrocardiographic parameters	Time after operation					Time after operation					
	Sham (n = 6)					I/R (n = 10)					*p*
	Baseline	6 h	1 day	3 days	7 days	Baseline	6 h	1 day	3 days	7 days	I/R vs. sham
SCL (ms)	121 ± 5	115 ± 8	110 ± 7	120 ± 9	123 ± 11	120 ± 8	106 ± 9^	104 ± 10^	107 ± 14^	**104 ± 11**^**	> 0.999/0.330/0.687/0.087/0.001
HR (bpm)	497 ± 20	522 ± 35	547 ± 43	504 ± 42	491 ± 51	502 ± 33	569 ± 48^	584 ± 57^	568 ± 74^	**582 ± 60**^**	> 0.999/0.334/0.587/0.083/0.003
P duration (ms)	22.9 ± 1.4	22.7 ± 2.4	21.6 ± 1.4	22.5 ± 2.0	24.1 ± 2.7	23.6 ± 1.8	25.0 ± 3.7	22.2 ± 1.9^	22.6 ± 2.4	22.7 ± 1.7	0.998/0.301/0.989/> 0.999/0.758
PR (ms)	35.1 ± 2.6	33.9 ± 2.7	34.1 ± 1.9	35.8 ± 1.7	36.5 ± 2.2	35.9 ± 1.7	34.5 ± 2.8	32.3 ± 2.0^	33.3 ± 2.2	33.8 ± 2.7	0.997/0.983/0.508/0.166/0.108
QRS (ms)	12.1 ± 0.9	10.9 ± 0.7	11.6 ± 0.8	12.1 ± 0.8	12.1 ± 1.1	11.7 ± 0.7	**13.8 ± 1.5**^***	**14.5 ± 1.3**^***	**15.9 ± 1.8**^***	**15.8 ± 1.9**^***	0.993/< 0.001/< 0.001/< 0.001/< 0.001
R amplitude (mV)	1.4 ± 1.0	1.8 ± 1.3	1.4 ± 1.1	1.3 ± 1.1	1.3 ± 0.7	1.4 ± 1.0	**0.5 ± 0.3**^**	**0.2 ± 0.2**^*	**0.3 ± 0.2**^	**0.3 ± 0.3**^	> 0.999/0.005/0.011/0.051/0.073
QT (ms)	55.0 ± 4.4	58.2 ± 5.1	58.5 ± 6.0	61.0 ± 4.3	56.5 ± 4.3	52.6 ± 2.9	61.9 ± 4.2^	**66.4 ± 5.6**^*	**68.5 ± 8.0**^*	**65.5 ± 6.1**^**	0.937/0.646/0.029/0.040/0.008
QTc (ms)	50.1 ± 3.7	54.4 ± 4.5	56.0 ± 5.8^	55.9 ± 4.5^	51.1 ± 4.6	47.8 ± 2.1	**60.1 ± 2.5**^*	**65.3 ± 3.1**^***	**66.2 ± 4.5**^***	**64.1 ± 4.3**^***	0.797/0.030/< 0.001/< 0.001/< 0.001
JT (ms)	42.9 ± 4.1	47.4 ± 4.7	47.0 ± 5.6	48.9 ± 4.1	44.3 ± 4.1	40.8 ± 2.4	48.2 ± 4.6^	51.9 ± 6.3^	52.7 ± 8.5^	49.7 ± 5.1^	0.951/0.999/0.328/0.608/0.236
JTc (ms)	39.0 ± 3.7	44.1 ± 4.2	44.8 ± 5.3	44.8 ± 4.3	40.0 ± 4.5	37.2 ± 1.9	46.7 ± 3.0^	50.9 ± 4.2*^	50.7 ± 5.5*^	**48.7 ± 3.6**^***	0.927/0.723/0.023/0.030/< 0.001
Tpeak-Tend (ms)	24.4 ± 2.4	25.0 ± 2.8	23.2 ± 4.5	25.3 ± 3.7	23.6 ± 3.2	21.5 ± 1.8	23.6 ± 4.1	27.6 ± 5.9^	26.9 ± 7.4^	26.5 ± 5.9	0.741/0.985/0.343/0.974/0.744

Data are given as mean ± SD. Statistical significance of differences between I/R and sham control was assessed by two-way ANOVA (Sidak); *p < 0.05; **p < 0.01; ***p < 0.001. The change of parameters after incident compared to baseline of each group was assessed by two-way ANOVA (Dunnett); ^*p* < 0.05.

Significant values are in bold.

Six-lead ECG recording during EPS in anesthetized mice confirmed the prolonged ventricular depolarization and repolarization intervals similar to those in telemetry (data not shown). Interestingly, fQRS was frequently observed in 15 out of 18 I/R mice (83%), which was completely absent in sham-operated mice (0%) (Fig. 1 and S2). There was no difference in VRP between I/R mice and sham mice. Detailed parameters are given in Table S1.

#### Enhanced ventricular arrhythmogenesis after I/R—telemetry

**Figure 2:**
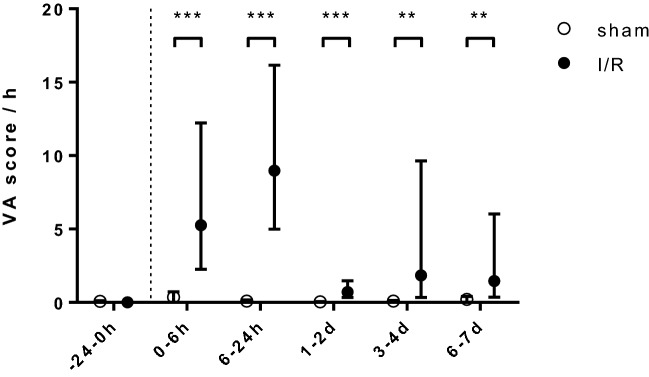
The susceptibility for spontaneous VA increases in the subacute stage after I/R. The herein applied arrhythmia score rates both number and severity of VA (see “Methods” section). VA score values of one hour are averaged at each given time interval. Data are given as geometric mean and 95% confidence interval (BL, *p* = 0.393; 0–6 h; ***p < 0.001; 6–24 h; ***p < 0.001; 1–2 days; ***p < 0.001; 3–4 days; **p = 0.003; 6–7 days; **p = 0.004; sham n = 6, I/R n = 10, non-parametric Mann Whitney U-test).

Spontaneous VA arose predominantly during the subacute stage after myocardial I/R (Fig. 2). Few VA were already observed during the surgical open-chest I/R procedure (Fig. S1). Infrequently, sham-operated mice showed single PVC without clustering during the entire observation period. As early as 6 h after I/R, however, VA occurred robustly. The VA score, which reflects the number and severity of VA, peaked out within the first 24 h after I/R. From days 3 to 4 and from days 6 to 7 after I/R, VA accumulated again (Fig. 2). Infarcted mice with fQRS did not exhibit more spontaneous VA compared to mice without fQRS (VA score; 0–6 h, 51 ± 49 vs 47 ± 39; 6–24 h, 181 ± 103 vs 297 ± 308; 1–2 days, 21 ± 13 vs 32 ± 34; 3–4 days, 120 ± 226 vs 784 ± 1211; 6–7 days, 66 ± 103 vs 361 ± 512).

#### Enhanced ventricular arrhythmogenesis after I/R—EPS

**Figure 3:**
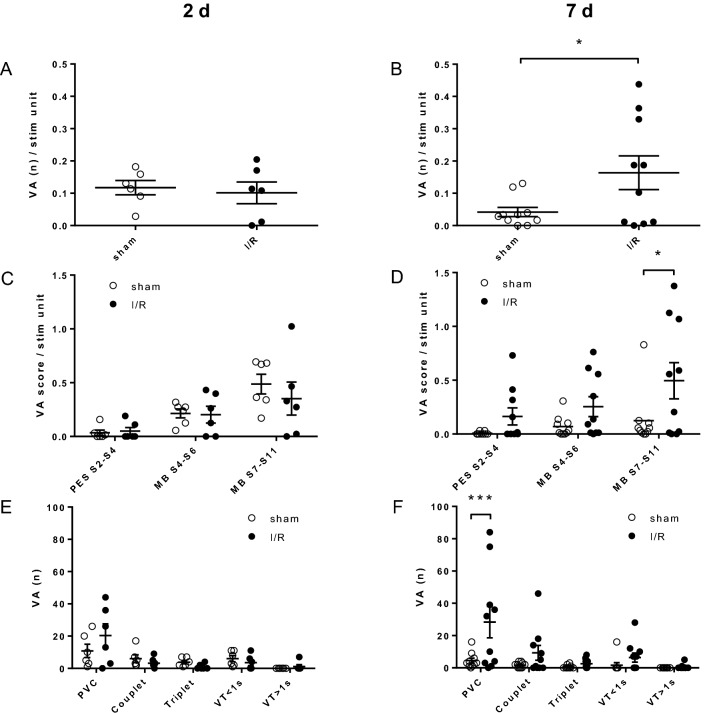
I/R raises the electrical ventricular vulnerability. (**A**) EPS reveals a similar burden of VA at 2 days after I/R compared to sham operated controls (*p* = 0.731, n = 6, unpaired two-sided *t*-test). (**B**) The number of VA inductions increases significantly 7 days after I/R over sham controls (*p* = 0.038, n = 10, unpaired two-sided *t*-test). (**C**) The severity of VA at 2 days is indifferent between groups independent of the used PES protocol (two-way ANOVA (Sidak); S2–S4, *p* = 0.998; S4–S6, *p* = 0.999; S7–S11, *p* = 0.596; n = 6). (**D**) Application of multiple extrastimuli is most efficient to elicit VA at 7 days post I/R over sham control (MB S7–S11, *p = 0.018, n = 10, two-way ANOVA (Sidak); PES S2–S5, *p* = 0.544; MB S4–S6, *p* = 0.404). (**E**) The number of induced VA classified by type did not differ at 2 days (two-way ANOVA (Sidak); premature ventricular complex (PVC), *p* = 0.130; couplet, *p* = 0.969; triplet, *p* = 0.969; VT < 1 s, *p* = 0.982; VT > 1 s = 0.999; n = 6). (**F**) Predominantly, more PVC are induced in I/R mice compared to sham operated controls (***p < 0.001, n = 10, two-way ANOVA (Sidak); couplet, *p* = 0.531; triplet, *p* = 0.998; VT < 1 s, *p* = 0.884; VT > 1 s, *p* > 0.999). All data are given as individual scatter dot plot and mean ± SEM.

2 days after I/R, PES induced VA in both the test and control groups to a similar extent (Fig. 3A,C). At 7 days however, I/R mice showed a significantly higher inducibility of VA compared to that of sham controls (Fig. 3B; *p* = 0.038). A recently proposed PES protocol proved more effective than conventional protocols at 7 days post I/R (Fig. 3D). Thus, application of multiple extrastimuli was most effective to sort out ventricular vulnerability in I/R over sham mice: it was best appreciated by induction of PVC at 7 days post I/R (Fig. 3F), whereas at 2 days, induced VA did not differ between groups (Fig. 3E). Moreover, at 7 days, VT were elicited in 5 out of 10 I/R mice and in only a single sham-operated mouse (*p* = 0.05).

**Figure 4:**
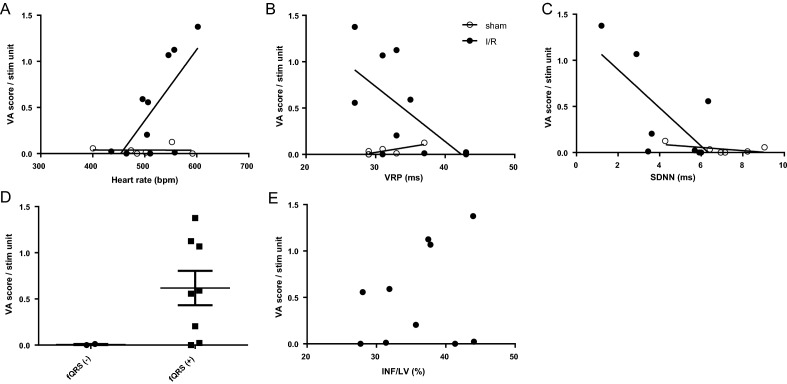
Electrical ventricular vulnerability is associated with sympathetic activation after I/R. (**A**) The induction of VA at 7 days after I/R is associated with heart rate (HR) (linear regression; I/R (filled circles): y = 0.007719*x − 3.503, r^2^ = 0.5011, *p* = 0.020); sham (open circles): y = − 9.577e−006*x + 0.04268, r^2^ = 0.0001752, n.s.), (**B**) with the ventricular refractory period (VRP) at 90 ms (linear regression; I/R (filled circles): y = − 0.05940*x + 2.515, r^2^ = 0.4022, *p* 0.048; sham (open circles): y = 0.01225*x − 0.3459, r^2^ = 0.6677, *p* = 0.047), and (**C**) with the standard deviation of normals to normals (SDNN) (linear regression; I/R (filled circles), y = − 0.2074*x + 1.314, r^2^ = 0.5032, *p* = 0.043; sham (open circles): y = − 0.01721*x + 0.1585, r^2^ = 0.3475, *p* = 0.218). (**D**) The induction of VA tends to be increased in I/R mice exhibiting fragmentation of the QRS complex (fQRS (+)) compared to those without fQRS (-) (unpaired one-sided *t*-test (Mann–Whitney), *p* = 0.066). (**E**) The severity of VA induction as indicated by the VA score did not significantly correlate with increasing infarct size (linear regression, y = 0.02416*x − 0.3725, r^2^ = 0.07636, *p* = 0.439). Data are given as mean VA score per stimulation unit. Statistical significance of differences between the slopes of the linear regression analyses was assessed by analysis of covariance using the F test: A (*p* = 0.029), B (*p* = 0.209), C (*p* = 0.112).

Inducible animals had higher HR (*p* = 0.020), shorter VRP (*p* = 0.043), and lower SDNN (*p* = 0.043), indicating enhanced sympathetic activity in I/R mice (Fig. 4A–C). Unlike with the occurrence of spontaneous VA, infarcted mice with fQRS tended to show increased ventricular inducibility in comparison to those without fQRS (VA (n)/stim unit; 0.618 ± 0.527 vs. 0.006 ± 0.008, *p* = 0.066; n = 8 vs 2) (Fig. 4D). The severity of VA did not correlate with IS (linear regression of VA on IS, y = 0.02416*x − 0.3725, r^2^ = 0.07636, *p* = 0.439) (Fig. 4E).

### I/R disturbs cardiac autonomic activity

**Figure 5:**
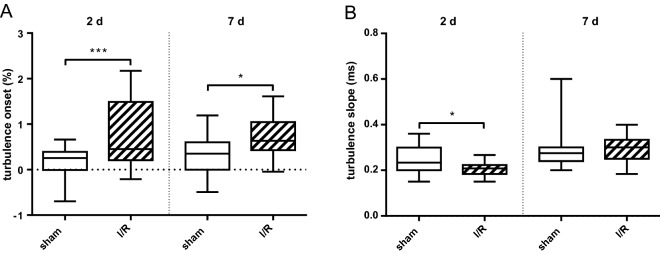
Baroreflex sensitivity is reduced after I/R. (**A**) Turbulence onset increases both at 2 days and 7 days after I/R as indicated (***p < 0.001 and *p = 0.046, respectively). (**B**) Turbulence slope is depressed at 2 days after I/R (*p = 0.034). The heart rate variability parameters turbulence onset and slope are surrogate markers for baroreflex sensitivity (see “Methods” section). Note that the results indicate a disturbed vagal nerve activity after I/R. Data are given as box and whiskers min to max (n = 6). Statistical significance of differences between I/R and sham control was assessed by a two-way ANOVA (Tukey’s multiple comparison).

I/R mice had higher HR compared to baseline at all the time points examined after injury. In sham-operated mice, HR did not significantly increase from baseline. HRV analysis indicated an impairment of time- and frequency-domain parameters after I/R (Table 3). Most parameters, except for the LF/HF ratio, were depressed after I/R; particularly, SDNN and SD2 were reduced in I/R mice until 7d after I/R. HR and parasympathetic activity were not associated with IS (linear regression of HR on IS, y = 4.629*x + 413.6, r^2^ = 0.2402, *p* = 0.217; linear regression of SDNN on IS, y = − 0.1074*x + 8.279, r^2^ = 0.1489, *p* = 0.345). Sham-operated mice showed only modest postoperative variations in HRV parameters, preponderantly without any statistical significance. Electrical induction of PVC by QRS-sensed pacing was employed to evaluate HRT. TO was markedly disturbed in I/R mice compared to sham mice, both at 2 days (*p* < 0.001) and 7 days (*p* = 0.046) post-I/R, respectively. TS was reduced 2 days after I/R vs sham (*p* = 0.034), but not on day 7 (Fig. 5). Altogether, these results indicate a disturbed baroreflex sensitivity in I/R mice with relevant changes early following reperfusion.

**Table 3:** Parasympathetic activity is diminished during first week after I/R.

HRV parameters	Time after operation					Time after operation					
	Sham (n = 6)					I/R (n = 10)					*p*
	Baseline	6 h	day 1	day 3	day 7	Baseline	6 h	day 1	day 3	day 7	I/R vs. sham
RR (ms)	122 ± 4	115 ± 7	110 ± 7	120 ± 9	124 ± 11	121 ± 8	107 ± 10^	103 ± 10^	108 ± 14^	**103 ± 11**^***	> 0.999/0.413/0.705/0.098/< 0.001
HR (bpm)	496 ± 15	526 ± 31	551 ± 34	505 ± 42	490 ± 47	501 ± 33	568 ± 49^	586 ± 59^	567 ± 75^	**593 ± 65**^**	0.999/0.442/0.634/0.107/0.001
SDNN (ms)	8.2 ± 1.7	7.8 ± 2.4	6.4 ± 1.8	6.7 ± 2.7	7.0 ± 1.6	8.8 ± 2.4	**4.1 ± 1.5**^***	**2.7 ± 1.0**^***	**3.1 ± 0.9**^**	**4.3 ± 1.7**^*	0.966/< 0.001/< 0.001/0.001/0.020
RMSSD (ms)	3.7 ± 1.2	4.3 ± 1.5	3.0 ± 1.0	2.7 ± 1.0	4.2 ± 1.8	5.2 ± 1.0	2.7 ± 1.6^	1.9 ± 0.8^	2.2 ± 0.8^	3.4 ± 1.8^	0.131/0.093/0.413/0.964/0.763
pNN6 (%)	13.5 ± 12.4	14.7 ± 11.8	5.9 ± 5.8	5.8 ± 5.1	17.4 ± 13.9	24.0 ± 10.5	6.5 ± 13.8^	1.1 ± 2.0^	3.2 ± 4.2^	10.6 ± 10.7^	0.191/0.439/0.883/0.990/0.632
Total power (ms^2^)	47.6 ± 19.4	60.7 ± 44.8^	43.6 ± 37.0	46.6 ± 30.5	58.8 ± 32.1	79.0 ± 41.9	**19.8 ± 14.8**^*	8.9 ± 5.4^	11.0 ± 6.5^	30.8 ± 21.5	0.129/0.022/0.073/0.062/0.222
VLF power (ms^2^)	32.3 ± 14.4	40.1 ± 31.9	29.0 ± 23.0	34.8 ± 28.0	33.6 ± 16.6	47.1 ± 38.8	**8.7 ± 5.9**^*	4.7 ± 4.3^	**4.6 ± 3.5**^*	6.4 ± 3.8^	0.580/0.017/0.107/0.024/0.053
LF power (ms^2^)	9.6 ± 6.2	12.0 ± 8.4^	10.5 ± 10.7	9.1 ± 5.5	16.1 ± 11.8	19.1 ± 7.9	6.6 ± 6.8^	2.1 ± 1.4^	3.8 ± 2.2^	14.6 ± 11.9	0.099/0.627/0.190/0.658/0.997
HF power (ms^2^)	5.9 ± 5.3	8.7 ± 7.0	4.3 ± 4.4	2.8 ± 2.3	9.7 ± 7.6	13.2 ± 7.8	4.6 ± 5.9^	2.0 ± 1.5^	2.6 ± 2.2^	10.1 ± 10.0	0.109/0.668/0.956/> 0.999/> 0.999
LF/HF ratio	2.3 ± 1.4	1.7 ± 0.6	2.8 ± 1.8	3.6 ± 2.9	2.3 ± 1.3	1.8 ± 0.9	1.7 ± 0.9	1.6 ± 1.0	2.0 ± 1.3	2.0 ± 1.2	0.966/> 0.999/0.442/0.145/0.997
SD1 (ms)	2.6 ± 0.8	3.0 ± 1.1	2.1 ± 0.7	1.9 ± 0.7	3.0 ± 1.2	3.7 ± 0.7	1.9 ± 1.1^	1.3 ± 0.6^	1.6 ± 0.6^	2.4 ± 1.2^	0.131/0.092/0.412/0.963/0.759
SD2 (ms)	11.3 ± 2.3	10.6 ± 3.2	8.8 ± 2.5	9.3 ± 3.7	9.4 ± 2.1	11.8 ± 3.6	**5.5 ± 2.0**^***	**3.6 ± 1.4**^***	**4.3 ± 1.3**^***	**5.5 ± 2.2**^*	0.994/< 0.001/< 0.001/< 0.001/0.014

*LF* low-frequency, *HF* high-frequency, *VLF* very low-frequency. Data are given as mean ± SD. Statistical significance of differences between I/R and sham control was assessed by two-way ANOVA (Sidak); *p < 0.05; **p < 0.01; ***p < 0.001. The change of parameters after incident compared to baseline of each group was assessed by two-way ANOVA (Dunnett); ^*p* < 0.05.

Significant values are in bold.

## Discussion

Murine animal models have proven particularly useful to study molecular mechanisms of diseases, which holds also true for cardiac arrhythmias as the leading cause of SCD in ischemic cardiomyopathy^15^. However, previously designed mouse models of MI to study VA seem limited in encompassing today’s clinical routine of early reperfusion therapy, as they have focused on chronic myocardial ischemia^11,12^. Here, we provide the first comprehensive and in depth hemodynamic, echocardiographic and electrophysiological in vivo characterization after reperfused MI in mice including established clinical markers of autonomic function and VA.

The main findings of our study demonstrate that myocardial I/R in mice (1) reliably increases ventricular arrhythmogenesis and susceptibility to VA, (2) delays depolarization and repolarization parameters in the ECG, and (3) depresses the cardiac parasympathetic tone. Thus, the presented animal model closely resembles functional sequelae of reperfused MI in humans after pPCI. It may thus prove useful to further investigate fundamental mechanisms of ventricular arrhythmogenesis after MI and to identify novel strategies in antiarrhythmic drug therapy.

### MI and electrocardiographic changes

The histological characteristics of our model of I/R, including AAR and IS, fully met the expectations for murine myocardial ischemia *in vivo*^10^. Echocardiography demonstrated impaired LV systolic function in the acute and subacute phase of one week after incident combined with a number of electrocardiographic findings, which show both similarities with and differences from previous studies of chronic cardiac ischemia in mice. LV dysfunction in I/R mice is typically less pronounced than in chronic MI mice^10,11^. However, direct comparison between the studies appears difficult, as different echocardiography parameters were taken to define LV function.

In our study, I/R mice presented with increased HR after surgery, which did not recover during the first week in contrast to sham-operated mice. I/R may thus have induced an imbalance in autonomic cardiac control. It cannot be excluded that also perioperative healing in I/R animals may have contributed to a temporary acceleration of HR over sham-operated controls. The previously mentioned studies of chronic MI in mice had shown only a short and transient increase in HR within the first 3 h after incident^11^. Lower HR within the first three days after MI were demonstrated in ECG tunnel experiments, potentially driven by analgesia using buprenorphine^7^. The ECGs we recorded from I/R mice showed widening of the QRS complex, similar in extent to what was reported for mice with chronic cardiac ischemia^12^. In addition, specific fQRS complex was documented in 14 out of 18 I/R mice. In contrast to humans, though, fQRS was not clearly associated with VA after acute MI in the small murine population we examined here^16^. Finally, I/R mice showed prolonged ventricular repolarization indices such as QT, QTc and JTc. Different from the study by Korte et al., we obtained these data from unrestrained freely moving mice by telemetric recordings, hence excluding putative effects of sedatives on cardiac excitability^12^. Prolonged repolarization may be explained by large voltage gradients between the infarct zone and the surrounding viable myocardium. The changes in the electrogram we observed did not correlate with IS, presumably due to the small size of the examined cohort.

### Increased ventricular vulnerability following MI

The here presented mouse model of I/R increases the susceptibility for VA. Except for early EPS, both spontaneous and induced VA scored significantly higher after I/R than in sham-operated control animals. Our results from mice correspond well with the demands for the current guidelines for the management of patients with VA: of spontaneous life-threatening VA occur most frequently within the first 24 h after acute MI and invasive risk stratification by PES early after MI may not be helpful for identification of high-risk VA patients^17^.

The two previous mouse models of permanent LAD ligation came to similar conclusions. However, the time course of VA evolvement seems different^11,12^: Gehrmann et al. detected an increased susceptibility for evoked VA only one week after chronic MI, whereas spontaneous VA were not detected within the first 14 days post-incident. Korte et al. describe an increased inducibility of VA at 11 weeks post-incident, accompanied by frequent spontaneous VA. Interestingly, we did not find any correlation between the occurrence of spontaneous VA and the susceptibility for and the severity of evoked VA. Thus, mice showing spontaneous VA did not develop a higher VA score in EPS, which was particularly evident within the early phase after I/R. Different underlying pathomechanisms may account for such discrepancy. Spontaneous VA are likely due to automaticity or triggered activity, whereas evoked VA will predominantly rely on re-entrant mechanisms involving the scarred myocardium^18^ and which are a conceivable substrate for VA induction in EPS. Thus, a complementary in-depth characterization of the arrhythmogenic substrate employing cardiac imaging and optical mapping techniques is needed. In summary, the here presented murine model of I/R may well be feasible to further assess post-ischemic VA, eventually even with respect to mechanism and risk stratification.

### Disturbed autonomic activity after MI

Our study demonstrates for the first time a profound relative decrease of parasympathetic tone after I/R in mice. Previous studies excluded a contribution of the cardiac autonomic nervous system to HR dynamics after MI^11,12^, well in line with the common understanding that mice normally exhibit a predominant sympathetic tone and a rather attenuated parasympathetic tone. Whereas HRV and HRT may be powerful predictors of mortality and SCD after MI in humans, two studies performed in rats and in mice were the only one up to date showing a decrease in HRV measures after no-reflow MI in rodents^7,19^. Here, HRV time-domain indices including SDNN were reduced in I/R mice, backed up by reduced frequency-domain indices like VLF and the non-linear measure SD2 during the whole first week after incident. In good agreement, HR was significantly increased in I/R mice and insufficiently recovered until 7 days after incident. We attribute the difference in results from our study compared to the previous ones in mice to at least the following aspects: (1) we provided the animals more time for recovery after transmitter implantation before recording baseline ECG, (2) we analyzed a larger and hence more representative cohort of animals, (3) we adapted our HRV analysis to more recent recommendations, and (4) we established an I/R model leading to a different quality of myocardial injury compared to permanent LAD ligation^4^.

It should be noted, however, that results from HRV analyses may also vary for the tested time intervals, animal characteristics, and a number of technical aspects. We have analyzed intervals of 3 min, for instance, whereas Gehrmann et al. chose shorter ones of 2 min and Korte et al. used 15 min. Moreover, Korte et al. reported much higher HR values (+ 200 bpm) in both ischemic as well as in sham-operated mice, eventually impeding the determination of parasympathetic activity. Manual verification of RR intervals is requisite for correct HRV analysis, as automated selection is often erroneous after I/R because of R-wave slurring. Finally, the updated HRV module we used in LabChart (ADInstruments) integrates optimized calculation of frequency-domain parameters using the Lomb-Scargle periodogram improving Fourier transformation as used by most others. Our findings of autonomic imbalance in HRV after I/R in mice are further supported by HRT analysis. Both TO and TS changed, indicating a blunted baroreflex sensitivity after I/R. Interestingly, a former study of myocardial cryolesion in mice showed TO abnormalities and TS reduction, which were also associated with an increased susceptibility for VT^20^. Altogether, the presented I/R model may prove suitable for further investigation of autonomic contribution to the emergence of VA after acute MI.

## Conclusion

The herein characterized animal model closely resembles functional sequelae of MI in humans under today´s clinical conditions including leading electrophysiological features and autonomic dysbalance. It may thus serve to further investigate fundamental mechanisms of ventricular arrhythmogenesis after MI and to identify novel strategies in antiarrhythmic therapy.

## Supplementary Information

Supplementary Legends.

Supplementary Information 2.

## Data Availability

The data generated and analyzed in this study are included in this article or may be obtained by the corresponding authors on reasonable request.
