# Supplementary material for: Cardiac ischemia and reperfusion in mice: a comprehensive hemodynamic, electrocardiographic and electrophysiological characterization
Source: Sci Rep. 2023 Apr 7;13:5693. doi: 10.1038/s41598-023-32346-5 (PMC10082073; doi:10.1038/s41598-023-32346-5)
Supplement: Supplementary file 1 — Supplementary Legends. [file 41598_2023_32346_MOESM1_ESM.docx]

**Supplemental Appendix**

Cardiac ischemia and reperfusion in mice: a comprehensive hemodynamic, electrocardiographic, and electrophysiological characterization

**Supplemental Figure Legends**

**Figure S1. First VA arise during I/R procedure.** VA score in anesthetized mice during open-chest surgery. During ischemia, VA occurred more frequently compared to sham operated mice (* p = 0.045). There was no difference between the ischemia and reperfusion phases (p = 0.290). Statistical significance of differences between experimental groups (sham: n = 16; I/R: n = 20) was assessed by one-way ANOVA (Kruskal-Wallis).

**Figure S2. Fragmented QRS in telemetric electrogram recordings is frequently observed after I/R.** Representative electrocardiograms from four mice (1 – 4) before and after I/R. The hallmark of I/R is QRS complex broadening and its deformation within the first 6 h after injury. The latter is characterized by an additional R wave or notching of the S wave (red circles).

**Supplemental Table**

**Table S1.** Heart rate behavior in response to catecholaminergic stimulation and ventricular refractory periods remain unchanged after I/R. Data are given as mean ± SD. Statistical significance of differences between I/R and sham control was assessed by one-way ANOVA (Tukey).
