# Supplementary material for: Cardiac ischemia and reperfusion in mice: a comprehensive hemodynamic, electrocardiographic and electrophysiological characterization
Source: Sci Rep. 2023 Apr 7;13:5693. doi: 10.1038/s41598-023-32346-5 (PMC10082073; doi:10.1038/s41598-023-32346-5)
Supplement: Supplementary file 2 — Supplementary Information 2. [file 41598_2023_32346_MOESM2_ESM.pdf]

Figure S1.

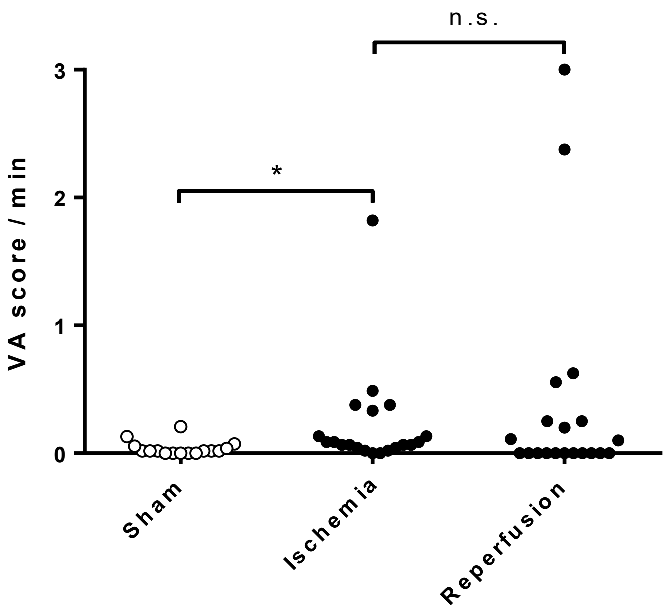

Figure S2.

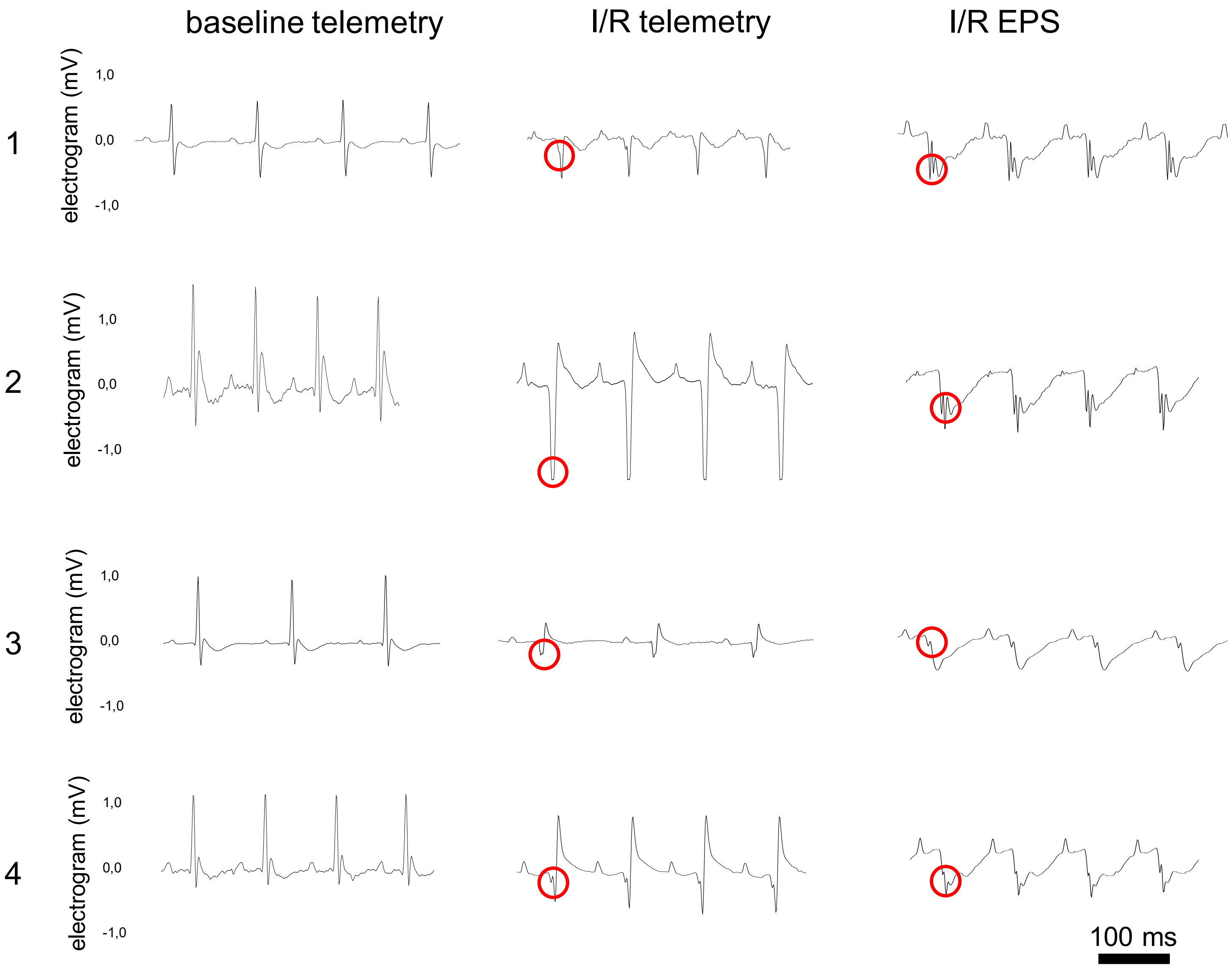

baseline telemetry
I/R telemetry
I/R EPS
1
2
3
4
electrogram (mV)
electrogram (mV)
electrogram (mV)
electrogram (mV)
1,0
0,0
-1,0
1,0
0,0
-1,0
1,0
0,0
-1,0
1,0
0,0
-1,0
100 ms

Table S1.

	sham 2 d<br>n = 6	sham 7 d<br>n = 10	I/R 2 d<br>n = 6	I/R 7 d<br>n = 10	p<br>I/R vs sham
Weight (g)	26 ± 1	28 ± 2	27 ± 2	27 ± 2	0.786 / 0.871
CL (ms)	122 ± 17	114 ± 12	117 ± 10	117 ± 12	0.917 / 0.988
HR (bpm)	501 ± 66	532 ± 58	516 ± 41	519 ± 51	0.962 / 0.984
HR Isoprenaline (bpm)	621 ± 45	604 ± 30	651 ± 59	626 ± 38	0.627 / 0.796
Delta HR Isoprenaline (%)	12 ± 5	12 ± 4	18 ± 14	11 ± 4	0.170 / 0.645
VRP 100 ms	27.0 ± 6.4	29.4 ± 3.0	28.3 ± 2.1	32.8 ± 5.7	0.999 / 0.999
VRP 90 ms	28.0 ± 6.8	30.0 ± 3.0	28.3 ± 1.6	34.0 ± 5.7	0.999 / 0.997
VRP 80 ms	28.0 ± 6.4	30.0 ± 3.4	29.0 ± 3.3	34.4 ± 6.9	0.999 / 0.991
